# Currently Available Strategies for Target Identification of Bioactive Natural Products

**DOI:** 10.3389/fchem.2021.761609

**Published:** 2021-09-30

**Authors:** Gen Li, Xuling Peng, Yajing Guo, Shaoxuan Gong, Shijie Cao, Feng Qiu

**Affiliations:** ^1^ School of Chinese Materia Medica, Tianjin University of Traditional Chinese Medicine, Tianjin, China; ^2^ Tianjin State Key Laboratory of Modern Chinese Medicine, Tianjin University of Traditional Chinese Medicine, Tianjin, China

**Keywords:** natural product, target identification, probe, non-probe, drug discovery

## Abstract

In recent years, biologically active natural products have gradually become important agents in the field of drug research and development because of their wide availability and variety. However, the target sites of many natural products are yet to be identified, which is a setback in the pharmaceutical industry and has seriously hindered the translation of research findings of these natural products as viable candidates for new drug exploitation. This review systematically describes the commonly used strategies for target identification via the application of probe and non-probe approaches. The merits and demerits of each method were summarized using recent examples, with the goal of comparing currently available methods and selecting the optimum techniques for identifying the targets of bioactive natural products.

## Introduction

Natural products (NPs) are a group of diverse and naturally-occurring chemical compounds or substances with a wide range of biological activities. NPs are considered as a vital source for new drug development that greatly assisted the field of drug innovation (Newman and Cragg, 2016). Recently, pharmaceutical companies and drug discovery organizations have identified a large number of bioactive molecules from NPs (Rodrigues et al., 2016), but the targets of action of these NP are still unidentified and the underlying mechanisms of action are unclear. Generally, the development of new drugs involves designing drug molecules based on their specific targets of action. Therefore, identifying the targets of bioactive NPs is essential for elucidating their mechanisms of action and optimizing existing drugs for hastening the process of new drug development (Lo et al., 2015; El-Wakil et al., 2017). A drug target refers to the specific site in which the drug binds to the biomolecules in the body and produces the desired therapeutic effect for the prevention and treatment of a specific disease (Zhang, 2012). Traditional drug development was based on the principle of “one ingredient, one target, one disease,” which indicates that the drug combines with a specific target to treat a particular disease. However, it is very common for drugs to combine with multiple targets (Klessig et al., 2016; Peon et al., 2017; Majumder et al., 2018), which can significantly interfere with target identification and isolation. Interestingly, this offers novel opportunities and possibilities for the discovery of new targets. Particularly for NPs with multiple effects and targets, the identification and elucidation of their corresponding targets of action may provide clearer interpretation and understanding of their biological properties (Zeng, 2018). A list of previously identified NPs, their specific drug targets, and location of discovery are presented in Table 1.

**TABLE 1:** Chemical structures, identification methods, specific drug targets, and biological applications of known natural products.

No.	Name	Chemical structure	Method	Specific target(s)	Location of discovery	Ref.
1	FK506 (Tacrolimus)	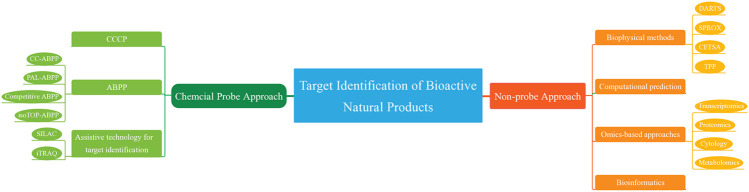	CCCP	FK506-binding protein (FKBP12), dynamin and neurocalc	Rat brain lysate	Mabuchi et al. (2015)
2	Radicicol	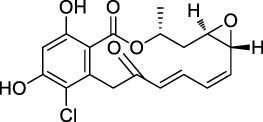	ABPP	Heat shock protein 90 (HSP90), ATP citrate lyase	HeLa cells	Ki et al. (2000)
3	LAF389	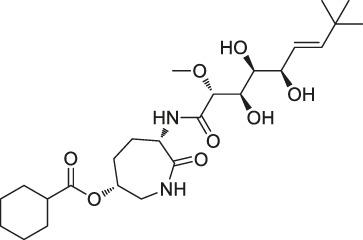	Proteomics	METAP	MDA-MB435 human breast cancer xenograft tumor	Towbin et al. (2003)
4	FR177391	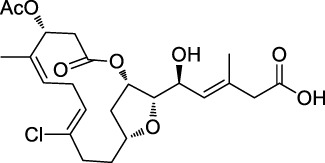	CCCP	Protein phosphatase 2A (PP2A)	3T3-L1 fibroblasts	Yamaoka et al. (2005)
5	Cyclosporin A	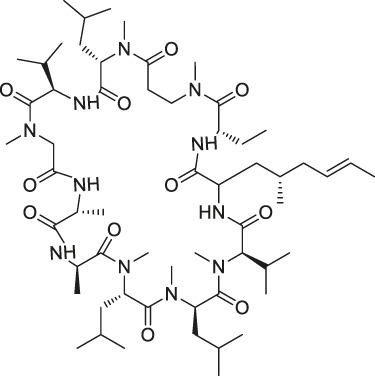	SPROX	Cyclophilin A (CYPA)	*Saccharomyces cerevisiae*	West et al. (2010)
			ABPP	Cyclophilin A (CYPA)	Protein mixture consisting of ovalbumin (OVA), carbonic anhydrase (CA), CYPA, and FK binding protein (FKBP)	Lamos et al. (2006)
6	Withaferin A	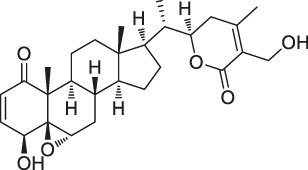	CCCP	Intermediate filament (IF) protein	Bovine aortic endothelial cells (BAECs)	Bargagna-Mohan et al. (2007)
7	Pateamine A	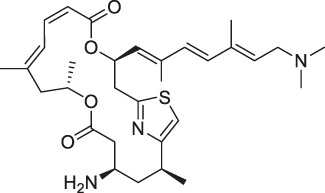	CCCP	Eukaryotic translation initiation factor 4A (eIF4A)	RKO cells	Low et al. (2007)
8	Marinopyrrole A	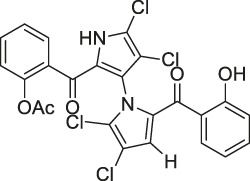 .	ABPP	Actin	HCT-116 cells	Hughes et al. (2009)
9	Resveratrol	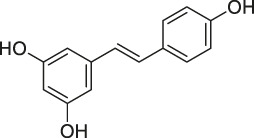	DARTS	elF4A	yeast strains	Lomenick et al. (2009)
10	Rapamycin	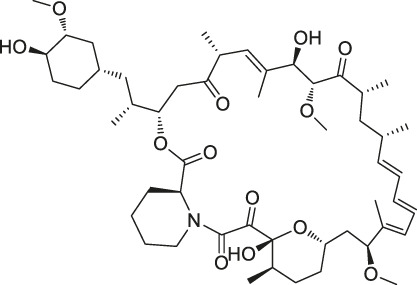	DARTS	FKBP12	*Bacillus subtilis*	Lomenick et al. (2009)
11	Showdomycin	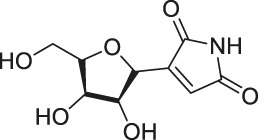	ABPP	Oxidoreductases and transferases	Pathogenic bacteria	Böttcher and Sieber (2010)
12	Vibralactone	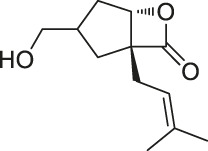	ABPP	Caseinolytic Clp protease (ClpP)	*Listeria monocytogenes*	Zeiler et al. (2011)
13	Vancomycin	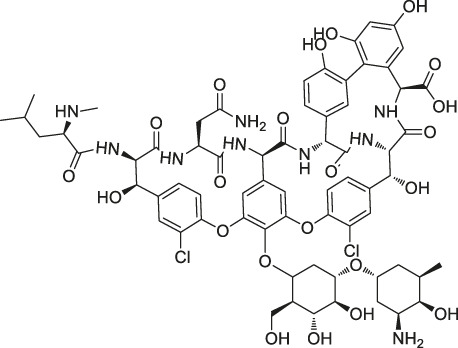	ABPP	Staphylococcal autolysin (Atl), ABC transporter protein	*Staphylococcus aureus* and *Enterococcus faecalis* strains	Eirich et al. (2011)
14	Staurosporine	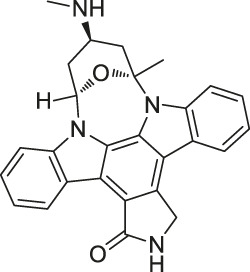	ABPP	Protein kinase A (PKA), c-Src, carboxyl-terminal Src kinase (CSK), Bruton’s tyrosine kinase (BTK), ESIw, non-protein kinases	HepG2 cancer cells	Shi et al. (2011)
15	Diosgenin	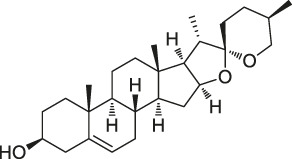	DARTS	1,25D3-MARRS/Pdia3/ERp57	5XFAD mice	Tohda et al. (2012)
16	Rugulactone	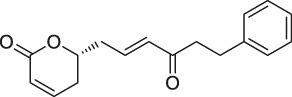	ABPP	Kinase THID	Pathogenic bacteria	Nodwell et al. (2012)
17	Duocarmycin	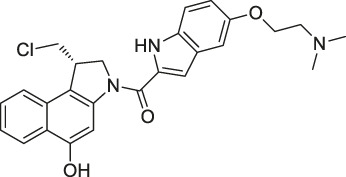	ABPP	Aldehyde dehydrogenase 1A1 (ALDH1A1)	A549 cancer cells	Wirth et al. (2012)
18	Celastrol	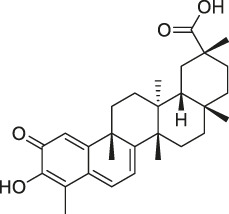	CCCP	Annexin II, eEF1A, β-tubulin	Human PANC-1 cells	Klaic et al. (2012)
19	Adenanthin	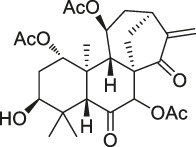	CCCP	Peroxiredoxin (Prx) I and Prx II peroxisomal cysteine (CP)	NB4 cells	Liu et al. (2012)
20	Eupalmerin acetate	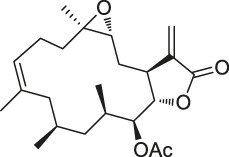	SILAC	Derlin 1 , cytochrome b5 , thromboxane A synthase 1	HL-60 leukemia cells	Li et al. (2013)
21	Hydroxyderricin	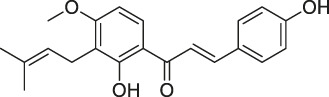	ABPP	Serine-tRNA synthetase	*Staphylococcus aureus*	Battenberg et al. (2013)
22	Hypothemicin	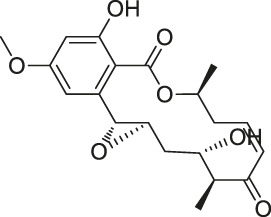	iTRAQ	Kinases (e.g., TbGSK3short, TbCLK1, TbCLK2)	*Trypanosoma brucei*	Nishino et al. (2013)
23	Oleocanthal	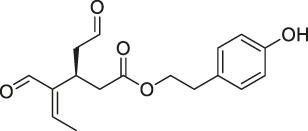	CCCP	HSP90	HeLa cells and histiocytic lymphoma (U937)	Margarucci et al. (2013)
24	Scalaradial	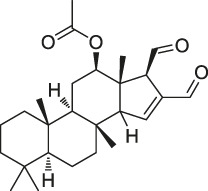	ABPP	PRXs, 14-3-3 soforms, proteasomes	HeLa cells	Cassiano et al. (2014)
25	Pyrethroid	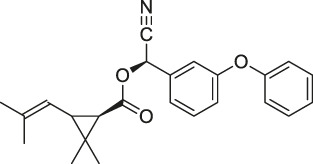	ABPP	Cytochrome P450 enzymes	Mouse liver microsomes	Ismail et al. (2016)
26	N-acetylaspartylglutamate (NAAG)	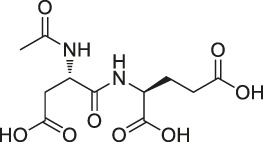	ABPP	Prostate-specific membrane antigen (PSMA)	Prostate cancer cells	Wang et al. (2014b)
27	Iberin	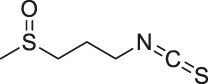	ABPP	Toll-like receptors (TLRs)	HEK293 cells expressing TLRs	Shibata et al. (2014)
28	Eriocalyxin	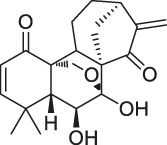	ABPP	Cysteine (Cys)62 of the p50 protein	SMMC-7721 HCC cells	Kong et al. (2014)
29	Cholesterol	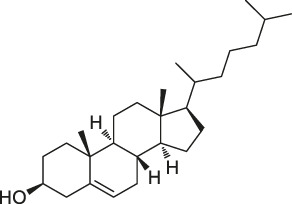	CCCP	Shh protein	HEK293a Shh^+^ cells	Ciepla et al. (2014)
30	Andrographolide	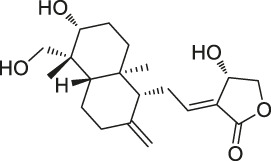	ABPP-iTRAQ	Multiple targets (e.g., Cys62 for NF-кB p50)	Human cancer cell lines	Wang et al. (2014a)
31	Acivicin	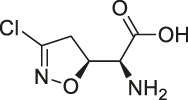	ABPP	ALDH4A1, carboxylesterase 1 (CES1)	Hepatoma cell lines, mouse liver tissue	Kreuzer et al. (2014)
32	Ainsliadimer A	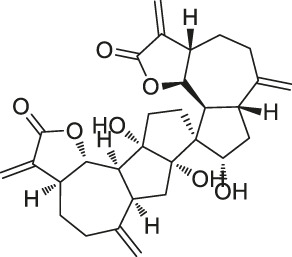	CCCP	Cys46 of IKKα/β	Mouse macrophage cell line RAW264.7	Dong et al. (2015)
33	Callyspongynic acid	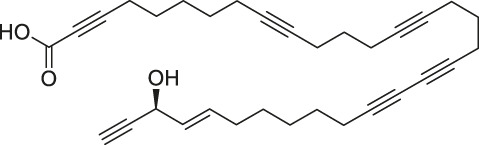	SILAC	Various membrane-associated proteins, lipid biosynthesis/metabolism-related proteins	HeLa cells, HEK293 cancer cells	Nickel et al. (2015)
34	Cerulenin	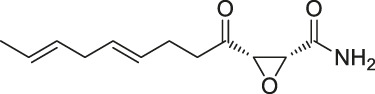	ABPP	Polyamine transporters (PATs)	Melanoma cells, HEK293 cells overexpressing PATase	Zheng et al. (2015)
35	Ecumicin	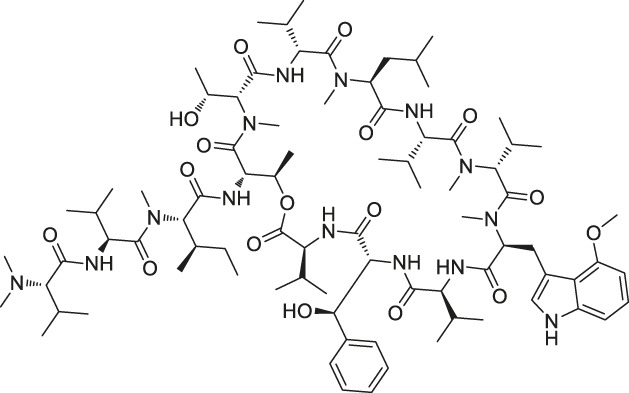	DARTS	ClpC1-ATPase complex	*Mycobacterium tuberculosis*	Gao et al. (2015)
36	Hydroxynonenal	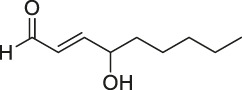	ABPP-SILAC	Multi-reactive Cys	RKO colon cancer cells	Yang et al. (2015)
37	Triptolide	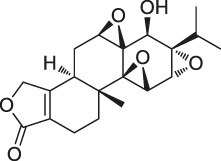	CCCP	Cys83, Cys173	MDCK cells	Zhao et al. (2015)
38	Zerumbone	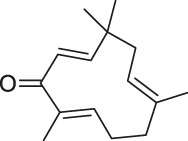	SILAC	Multiple proteins	HeLa cells	Kalesh et al. (2015)
39	Artesunate	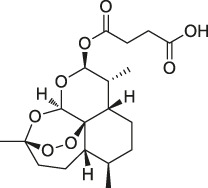	Proteomics	Protein JCHGC09008, *Plasmodium berghei* cytochrome oxidase	*Schistosoma japonicum*-susceptible mouse	Kong et al. (2015)
40	Chalcone	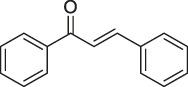	ABPP	β-microtubulin	A549 cells	Zhou et al. (2016a)
41	Folic Acid	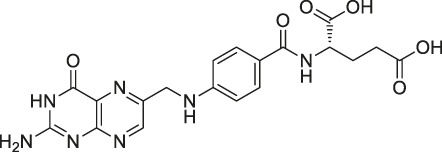	ABPP	Folate receptor α (FRα)	Fr-positive ovarian cancer phase II clinical trial	Srinivasarao et al. (2015)
42	Geldanamycin	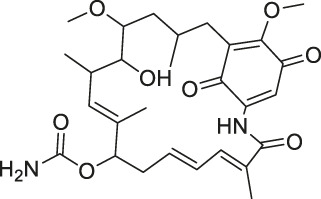	SPORX	HSP90	MCF-7 cells	Xu et al. (2016)
43	Manassantin A	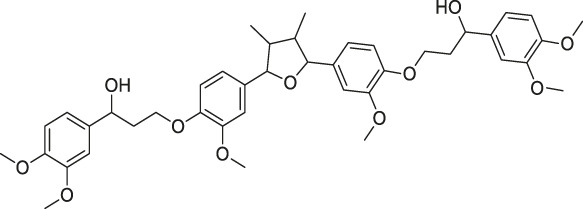	SPORX	Filamentin A, elongation factor 1α	MDA-MB-231 cells	Geer Wallace et al. (2016)
44	Daptomycin	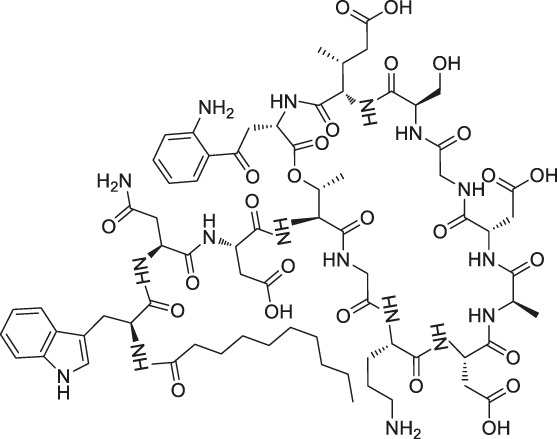	DRATS	Human ribosomal protein S19	HeLa cells	Gotsbacher et al. (2017)
45	Kongensin	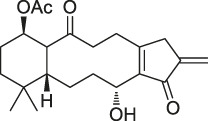	CCCP	HSP90, Cys420	HeLa-RIPK3 cells	Li et al. (2016)
46	Gambogic acid	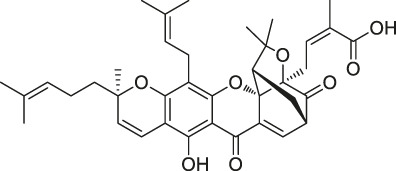	ABPP	Thioredoxin-related transmembrane protein 1 (TMX1), TMX2, transferrin receptor (TFRC), ribosomal protein S27a (RPS27A)	Activated HeLa cells, K562 cells	Zhou et al. (2016c)
47	Curcumin	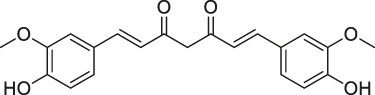	ABPP-iTRAQ	Multiple proteins	HCT116 colon cancer cell line	Wang et al. (2016)
48	Bile acid	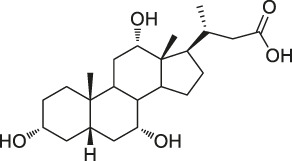	ABPP-SILAC	Takeda G protein-coupled receptor 5 (TGR5)	HeLa cells	Zhuang et al. (2017)
49	Naringenin	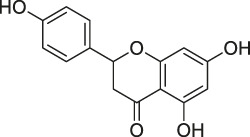	DARTS	Collagen response mediator protein 2 (CRMP2)	5XFAD mice	Yang et al. (2017)
50	Artemisinin	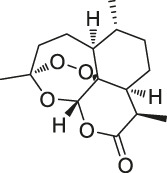	CCCP	Gephryin protein	Mouse β-cell line Min6	Li et al. (2017)
51	Betulinic acid	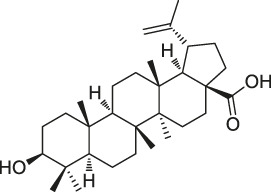	CCCP	Apoptosis-inducing factor mitochondrion-associated 1 (AIFM1), metadherin (MTDH), PDEX16	MCF-7 cells	Guo et al. (2017)
52	Matrinel	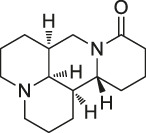	ABPP	Annexin A2	Hep3B cells (an HCC cell line prone to migration and invasion)	Wang et al. (2017a)
			DARTS	HSP90	SCI mice	Tanabe et al. (2018)
53	Pseudolaric acid B	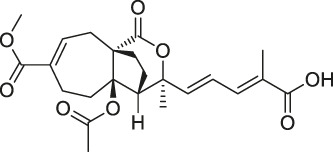	ABPP	Immunoglobulin C2 (IgC2)	Transmembrane protein CD147	Zhou et al. (2017)
54	Ramariolide	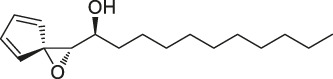	ABPP	30S ribosomal proteins S4 (RpsD) and S5 (RpsE), ClpX, Ask, Hsd	*Mycobacterium* cells	Lehmann et al. (2016)
55	Spongiolactone	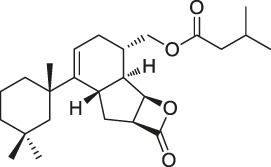	ABPP	Abhydrolase domain containing 10 (ABHD10), ABHD16A, neutral cholesterol ester hydrolase 1 (NCEH1)	K562 cells, leukemia T-cell line (Jurkat) cells	Wright et al. (2017b)
56	5-epi-Sinuleptolide	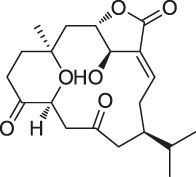	DARTS	Actin	Microtubules	Morretta et al. (2017)
57	Oridonin	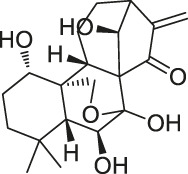	DARTS	Nucleophosmin	Jurkat cells, HeLa cells	Vasaturo et al. (2018)
58	Gephyronic acid	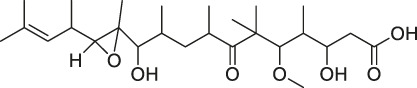	DARTS	eIF2α	Cancer-derived related cells	Rishi et al. (2018)
59	Arzanol	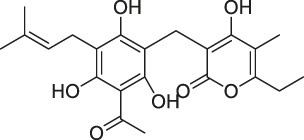	DARTS	Brain glycogen phosphorylase (BGP)	HeLa cells	Del Gaudio et al. (2018)
60	Quinine	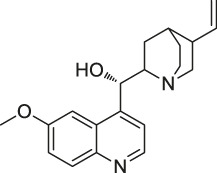	CETSA	Phosphorylase of purine nucleosides (PfPNP)	*Plasmodium falciparum*	Dziekan et al. (2019)
61	Vioprolide A	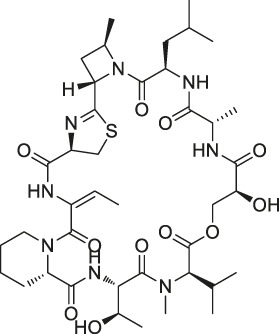	TPP	Nucleoporin 14 (NOP14)	Human acute lymphoblastic leukemia (ALL) cells	Kirsch et al. (2020)
62	NPD10084 (from the chemical library of RIKEN Natural Products Depository)	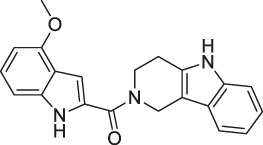	CETSA	Pyruvate kinase muscle isoform 2 (PKM2)	Colorectal cancer cells	Nagasawa et al. (2020)

CCCP, compound-lefted chemical proteomics; ABPP, activity-based protein profiling; DARTS, drug affinity responsive target stability; SPROX, stability of proteins from rates of oxidation; SILAC, stable isotope labeling with amino acids in cell culture; iTRAQ, isobaric tags for relative and absolute quantitation; CETSA, cellular thermal shift assay; TPP, thermal proteome profiling.

The target identification methods for NPs are generally classified into two strategies (Chen, 2016) : chemical probe and non-probe. Chemical probe approach includes Compound-centered chemical proteomics (CCCP) and Activity-based protein profiling (ABPP), while non-probe approach includes biophysics, Omics-based approaches and computational prediction using chemical biology data. Currently, chemical probe approaches are more commonly used than non-probe approaches. However, non-probe approaches have higher efficiencies and yields (Isgut et al., 2018). This review will systematically describe the currently available methods for target identification, summarize their advantages and disadvantages, and provide representative examples.

## Chemical Probe Approach

**FIGURE 1:**
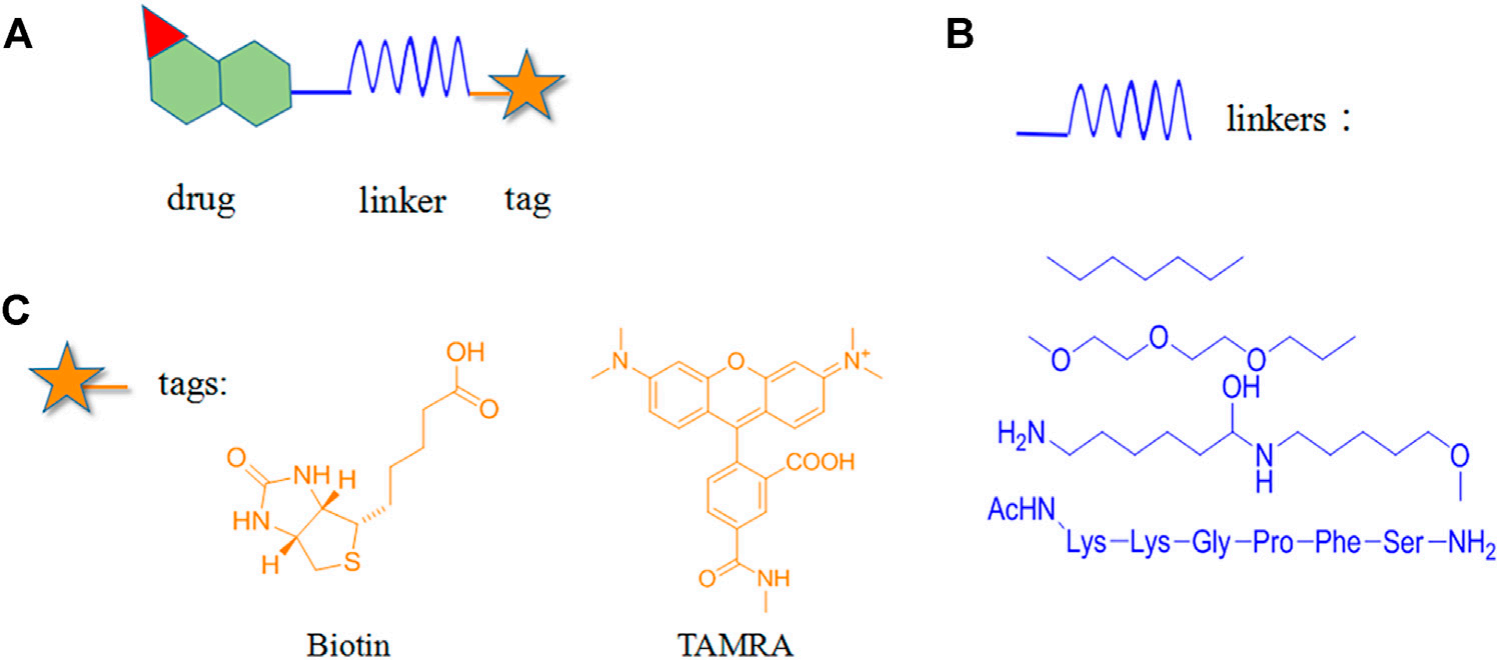
Structural composition of chemical probes. **(A)** Basic structure of chemical probes. **(B)** Example of commonly used linkers. **(C)** Example of commonly used reporter groups.

Chemical probe approach is a growing field using biology and chemistry for combining specific substances with NPs molecules to form probes. It has become a commonly used method for target identification as it can specifically identify target proteins without affecting their biological activity and function. The probes are generally composed of three components (Figure 1): 1) the active group is a structure with special biological activity in the NPs that can directly bind to the target protein (Chang et al., 2016) ; 2) the reporter group consists of the tag, which is used for rapid target–probe complex positioning, enrichment, and purification; and 3) the linker connecting the active and reporter groups, providing enough space for the two and ensuring that no interference with each other (Ma et al., 2018).

Depending on the binding site between the NPs and target protein, the reporter group may be composed of biotin, radio-labeled and fluorescent-labeled. Biotin is one of the most widely used reporter groups due to its strong binding capacity for streptavidin proteins. In practice, the NP is first modified and linked to the labelled biotin. Then, the NP is immobilized on a solid-phase carrier using the interaction between biotin and streptavidin protein. After co-culturing with the lysed cells and/or tissues for a certain period, the contact time between the NPs and target proteins in the cells or lysates is increased. Finally, a suitable lysis buffer is selected for elution, and the target protein is identified and isolated for enrichment. For example, Li’s group genetically modified a mouse β-cell line (Min6) to create a model capable of inducing aristaless related homeobox (ARX) overexpression. Min6 was co-cultured with solid-loaded artemisinin, which was found to bind to specific proteins in pancreatic islet α cells and activate γ-aminobutyric acid (GABA) receptors, inducing ARX displacement from the nucleus to the cytoplasm and thereby promoting the transformation of pancreatic islet α cells into pancreatic islet β cells. This study provided new insights for the treatment of type I diabetes (Li et al., 2017).

Furthermore, many research groups have used radio-labeled or fluorescent-labeled probes to identify the targets of a range of bioactive NPs, such as the flavonoid 7-O-cinnamoyl paclitaxel (Gunesch et al., 2020) , xanthohumol from hops (Brodziak-Jarosz et al., 2016) , and artemisinin (Yang J. et al., 2020) . Cephalosporin I, which was recently synthesized by Amatuni’s group using facile chemoenzymatic synthesis, exhibited selectivity for proteasome subunits β2 and β5 after the introduction of fluorescent labels. Further exploration of the conformational relationships revealed that macrocyclic seco-alcohols and the unsaturation and terminal branching of the lipid tail were essential for high inhibitory potency (Amatuni et al., 2020).

### Compound-Centered Chemical Proteomics

**FIGURE 2:**
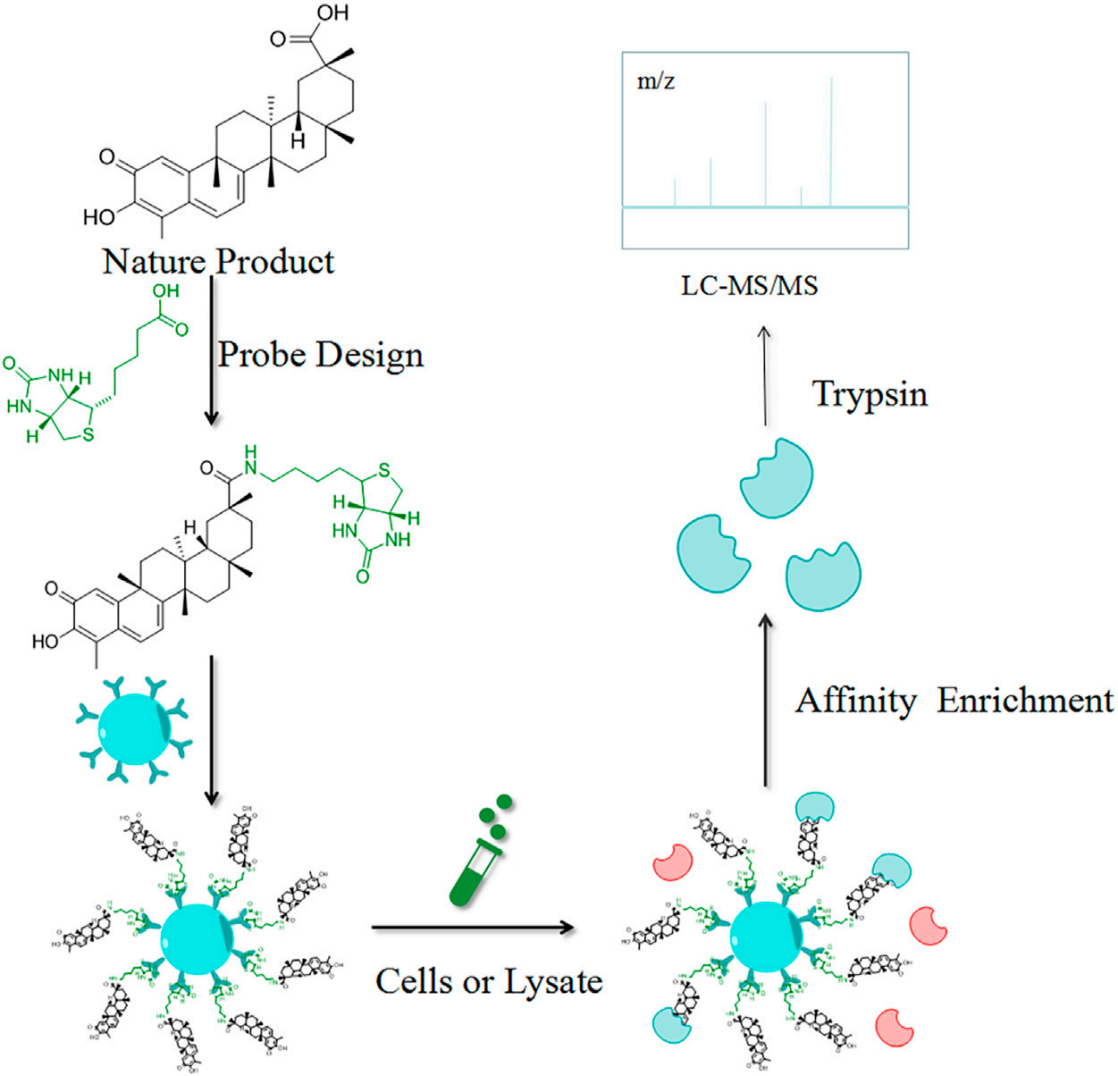
Schematic diagram of the target hooking technique. The NP is first structurally designed to be anchored to an insoluble support. Elution is performed after contact with the cell lysate, and the target proteins interacting with the affinity molecules are retained and identified by high-resolution mass spectrometry (MS).

CCCP is a simple and direct strategy for the identification of target proteins, which are isolated for enrichment based on their interactions with the NPs. As the most used CCCP method, the target hooking technique is based on the structure of the NPs by selecting certain NPs molecules to immobilize on an insoluble support, which is used to adsorb target proteins with specific affinity (Figure 2). Elution is performed after contact with the cell lysate, and the target proteins interacting with the affinity molecules are retained and identified by polyacrylamide gel electrophoresis (PAGE) and high-resolution mass spectrometry (HRMS) (Isgut et al., 2018). Harding et al. first used this method to isolate FKBP12, a binding protein of FK506 (Tacrolimus), and then Mabuchi et al. demonstrated that dynamin and neurocalc were also potential targets (Mabuchi et al., 2015). A variety of NPs targets have been identified using CCCP, including withaferin A (Bargagna-Mohan et al., 2007), handelin (Wang L.-C. et al., 2017), *Inula japonica* Thunb.(Liu et al., 2014), pateamine A (Low et al., 2007), triptolide (Zhao et al., 2015), celastrol (Klaic et al., 2012), sappanone (Liao et al., 2017) and kongensin A (Li et al., 2016). The non-covalent interaction between target proteins and NPs is key to the implementation of the CCCP strategy, and the reaction sites of both affect how the compounds are immobilized on the substrate. For instance, Margarucci et al. used HeLa and U937 cells as the model systems for solid and hematological tumor cell lines, respectively, and immobilized oleocanthal (OLC) by inserting spacer arms onto carbonyl bis-imidazole agarose beads. Experiment proved that HSP90 is a potential target for OLC (Margarucci et al., 2013).

Similarly, Guo et al. used CCCP to link a reporter group to betulinic acid (BA) and identified a potential target for its antitumor activity (Guo et al., 2017). Furthermore, Liu et al. demonstrated through CCCP that adenine targeted peroxiredoxin (Prx) I and Prx II to treat acute promyelocytic leukemia (Liu et al., 2012). Notably, Dong’s team used CCCP to identify the target of ainsliadimer A, which was discovered to exert anti-cancer and anti-inflammatory effects by acting on the cysteine of IKKα/β, and blocking the NF-κB signaling pathway (Dong et al., 2015).

CCCP, which combines the cross-cutting integration of synthetic chemistry, cell biology, and MS, provides the easy synthesis and indiscriminate analysis of all adsorbed proteins. However, this method has two shortcomings: one is that molecules with specific affinity are difficult to obtain and the other is the difficulty in immobilizing NPs with large and diverse molecular structures on solid-phase carriers while simultaneously retaining their activity. One possible approach to this is the method developed by Zeng’s group, which is to bond photosensitive groups to the solid-phase carrier, thus achieving the immobilization of the active molecules and obtaining the corresponding target groups (Zeng and Tu, 2017). Conventional target identification methods can only be performed *in vitro*, in which magnetic nanoparticles with smaller particle size are developed that can be selectively distributed into organs for *in vivo* target capture (Wang et al., 2019b). For example, Wang’s team used affinity-based ultrafiltration-high-performance liquid chromatography to directly identify the specific ligands for cytochrome P450 1A2, 3A4, and 2C9 in Danshen extract s (Wang Z. et al., 2018). In addition, the introduction of probes to the target hooking technique may have an impact on the identification of target proteins. Some NPs may have a change in phenotype or conformation due to excessive spatial resistance of the probe itself or may be introduced in an inappropriate location, thus affecting their biological activity and hindering their interaction with the corresponding target protein (Bachovchin et al., 2009).

### Activity-Based Protein Profiling

**FIGURE 3:**
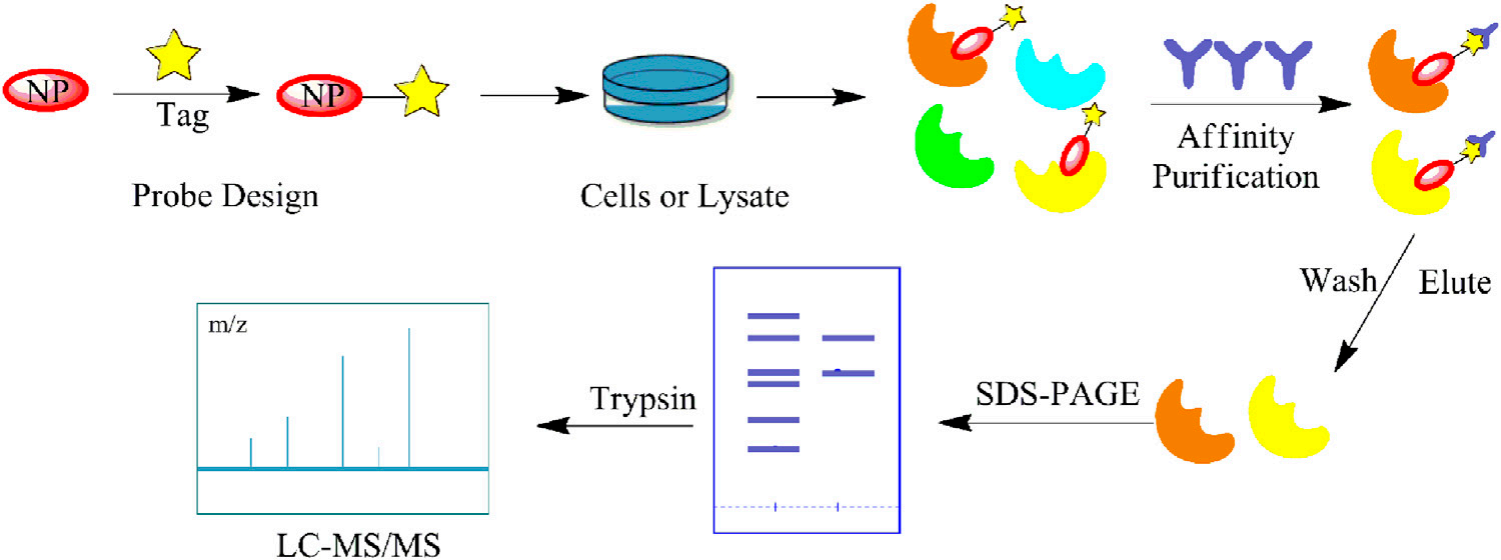
General workflow of the activity-based protein profiling (ABPP) method. The NP is first probed for specific affinity to adsorb the target protein. Affinity purification and elution are performed after contact with the cell lysate, and identified by sodium dodecyl sulfate (SDS)-PAGE and MS.

Although a relatively new strategy compared to CCCP, ABPP has become a well-established and stable method for target identification of bioactive NPs. The general workflow of ABPP is shown in Figure 3. Böttcher et al. reported the antibacterial effect of showdomycin against *Staphylococcus aureus* using this method (Böttcher and Sieber, 2010). Ciepla et al. synthesized an alkynyl sterol probe, an excellent cholesterol mimic, that effectively labelled the Sonic hedgehog (Shh) protein and allowed its visualization and analysis (Ciepla et al., 2014). Furthermore, using ABPP, Ken et al. discovered that radicicol can bind and inhibit the mammalian adenosine triphosphate (ATP) citrate lyase (Ki et al., 2000). Generally, ABPP employs reactive probes with reactive groups to bind and covalently modify the active site of a specific protein and determine its function. In 2007, Cravatt’s group first utilized ABPP to monitor the functional state of enzyme activity in complex biological systems (Barglow and Cravatt, 2007). The target proteins for vibralactone (Zeiler et al., 2011), vancomycin (Eirich et al., 2011), staurosporine (Shi et al., 2011), pyrethroid (Ismail et al., 2016), cerulenin (Zheng et al., 2015), folic acid (Srinivasarao et al., 2015), matrine (Wang D. et al., 2017), pseudolaric acid B (Zhou et al., 2017), spongiolactone (Wright et al., 2017b), NPs are identified using ABPP. Nodwell et al. used the Overman rearrangement and catalytic asymmetric esterification reaction for the synthesis of brassinolide, followed by the introduction of an alkyne handle into the structure (Nodwell et al., 2012). To keep the ABPP probe as structurally similar to rugulactone, the alkyne was directly attached to the C-16 position of the additional aromatic ring away from the Michael receptor in the molecule. The resulting ABPP probe was synthesized to validate the inhibitory effect of rugulactone, in which the 4-amino-5-hydroxymethyl-2-methylpyrimidine phosphate (HMPP) kinase was the main target (Nodwell et al., 2012). Additionally, Lehmann et al. discovered the effect of ramariolide on amino acid anabolism in *Mycobacterium avium* (Lehmann et al., 2016). Furthermore, Kong et al. demonstrated that NF-κB signaling in *Tricholoma tigrinum*-induced SMMC-7721 hepatocellular carcinoma cells can be inhibited by targeting the p50 protein (Kong et al., 2014). Scalarradial, a NP of marine origin with anti-inflammatory activity, was discovered to have peroxidase as its primary target by Cassiano et al. (2014). Currently, target proteins in complex systems can be investigated using ABPP in combination with other techniques, such as click chemistry-ABPP (CC-ABPP), photoaffinity labeling-ABPP (PAL-ABPP), competitive ABPP, and isotope tandem orthogonal proteolysis-ABPP (isoTOP-ABPP).

#### Click Chemistry-Activity-Based Protein Profiling

**FIGURE 4:**
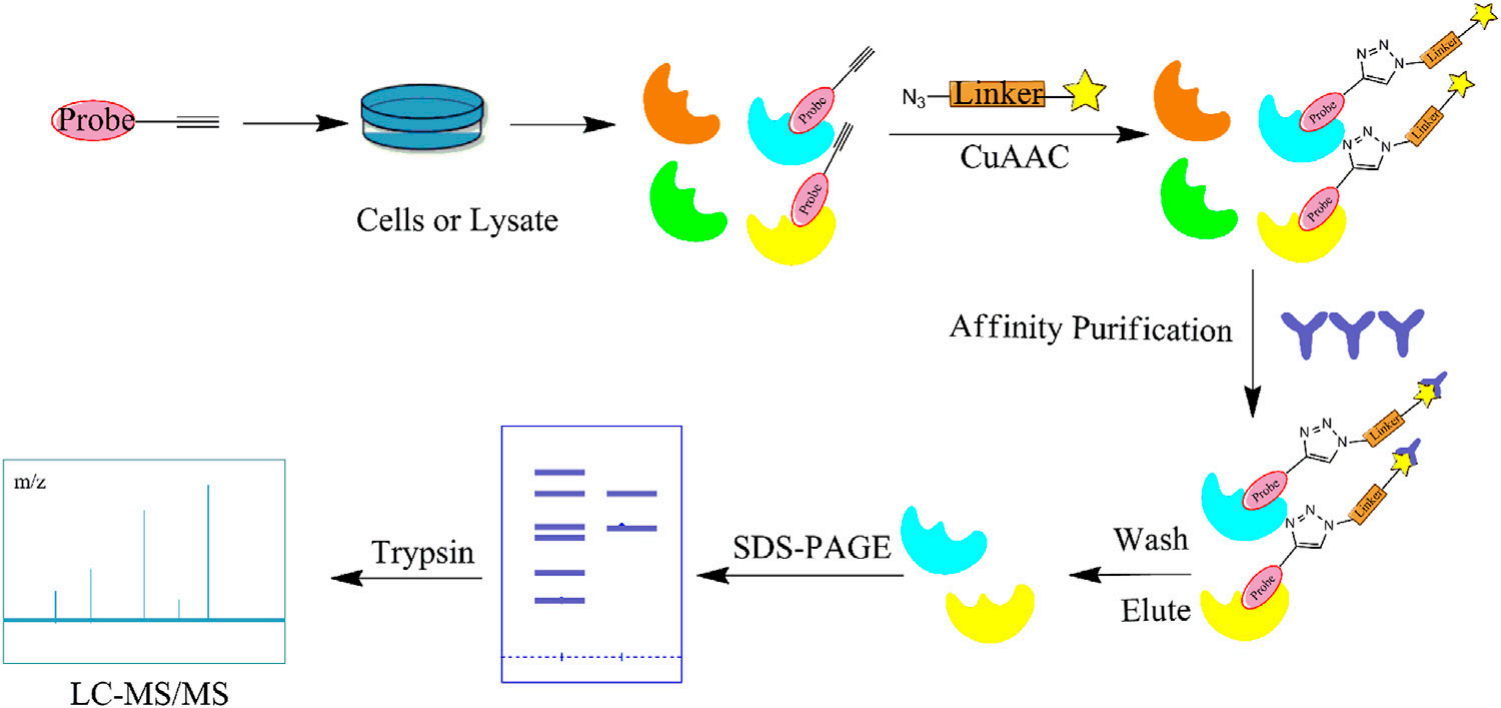
Flow diagram of the click chemistry–activity-based protein profiling (CC-ABPP) strategy. It starts with the synthesis of a NP with a terminal alkyne. After sufficient binding to the target protein, the probe is formed by a click reaction with an azide bearing a fluorescent or radioactive moiety. The target protein is subsequently identified by SDS-PAGE and MS.

In recent years, CC has become the main method for combination with ABPP due to its wide range of applications, lack of toxic by-products, and ability to produce reactions in aqueous solutions (Li et al., 2019a). CC-ABPP starts with the synthesis of a NP with a terminal alkyne, which is incubated with live cells. After sufficient binding to the target protein, the probe is formed by a click reaction with an azide bearing a fluorescent or radioactive moiety. The target protein is subsequently identified by sodium dodecyl sulfate (SDS)-PAGE. (Figure 4) In addition to the expected fatty acid synthase (FAS), eight new targets were identified. This experiment is the first to demonstrate the applicability of ABPP for identifying other unknown cellular targets. In addition, Zhou et al. identified β-microtubulin as the anticancer target of chalcone by introducing azide and alkyne groups to modify the probe C95 (Zhou B. et al., 2016), while Prothiwa et al. specifically labeled the active site of *Pseudomonas aeruginosa* quinolone biosynthetic enzyme PqsD using an α-chloroacetamide probe with a terminal alkyne, laying the foundation for the discovery of other enzyme inhibitors (Prothiwa and Böttcher, 2020). On the other hand, the antineoplastic drug acivicin has limited clinical application because of its inherent toxicity. However, Kreuzer et al. identified acetaldehyde dehydrogenase as the target of acivicin, offering the possibility of further exploring its cytotoxic effects (Kreuzer et al., 2014). Several NPs have also been identified as specific targets for pharmacological action using CC-ABPP, including gambogic acid (GA) (Zhou et al., 2016c), pseudolaric acid B(PAB) (Zhou et al., 2017), and quercetin and quercetin-4-O-β-glucoside (Shibata et al., 2014). The advantages of the CC-ABPP strategy include the detection of smaller sized probes, greater cell membrane penetration, and ability to complete the covalent reaction before cell disruption, allowing multiple modifications to the NPs without the need to develop new synthetic methods (Chen et al.) However, copper(I)-catalyzed azide–alkyne cycloadditions (CuAAC) can be cytotoxic because of the copper catalyst and cause considerable cell death, which is a major limitation in *in vivo* experiments. To reduce the cytotoxicity caused by copper, the Diels-Alder reaction (Devaraj et al., 2009) the reaction of tetrazine with cyclopropane (Patterson et al., 2012) is frequently used.

#### Photoaffinity Labeling-Activity-Based Protein Profiling

The CC-ABPP method is primarily applicable for the covalent binding of NPs to targets. However, the majority of NPs is actually bound to target proteins in a non-covalent and irreversible manner, and thus, are unstable. The combination of PAL and ABPP has reduced these restrictions and facilitated the identification of target proteins (Smith and Collins, 2015). The PAL-ABPP strategy mainly employs the addition of a photosensitive group to the original NP structure, which is activated under specific UV irradiation, prompting the NP to form a covalent linkage with the target protein and subsequently facilitating the enrichment and identification of the target protein. Benzophenone, aryl azide, and diazirine are the most commonly used photoaffinity groups for PAL-ABPP. Matthew’s group used diazirine as the photoaffinity group and terminal alkyne as the functional handle to synthesize a bioactive photoaffinity probe for actinomycin A by conveniently binding to the reporter group via a CuAAC reaction (Anketell et al., 2020). Luo et al. also used this label to study fruit extracts of *Ligustrum lucidum* Ait and discovered that 3-O-cis- or 3-O-trans-p-coumaroyl maslinic acid (OCMA) specifically acted on the S1 subsite of γ-secretase (Luo et al., 2020), while Lamos et al. identified multiple targets of action of cyclosporine using photosensitive moieties (Lamos et al., 2006). In addition, the PAL-ABPP strategy identified the human opioid daunorphan associated with antibacterial action (Wright et al., 2017a), LptA and LptD subunits in *Escherichia coli* periplasm (Vetterli et al., 2018). PAL-ABPP has also been applied for the interaction of the transcriptional regulatory protein AlgP in Gram-positive and -negative bacteria (Zhao et al., 2019).

#### Competitive Activity-Based Protein Profiling

**FIGURE 5:**
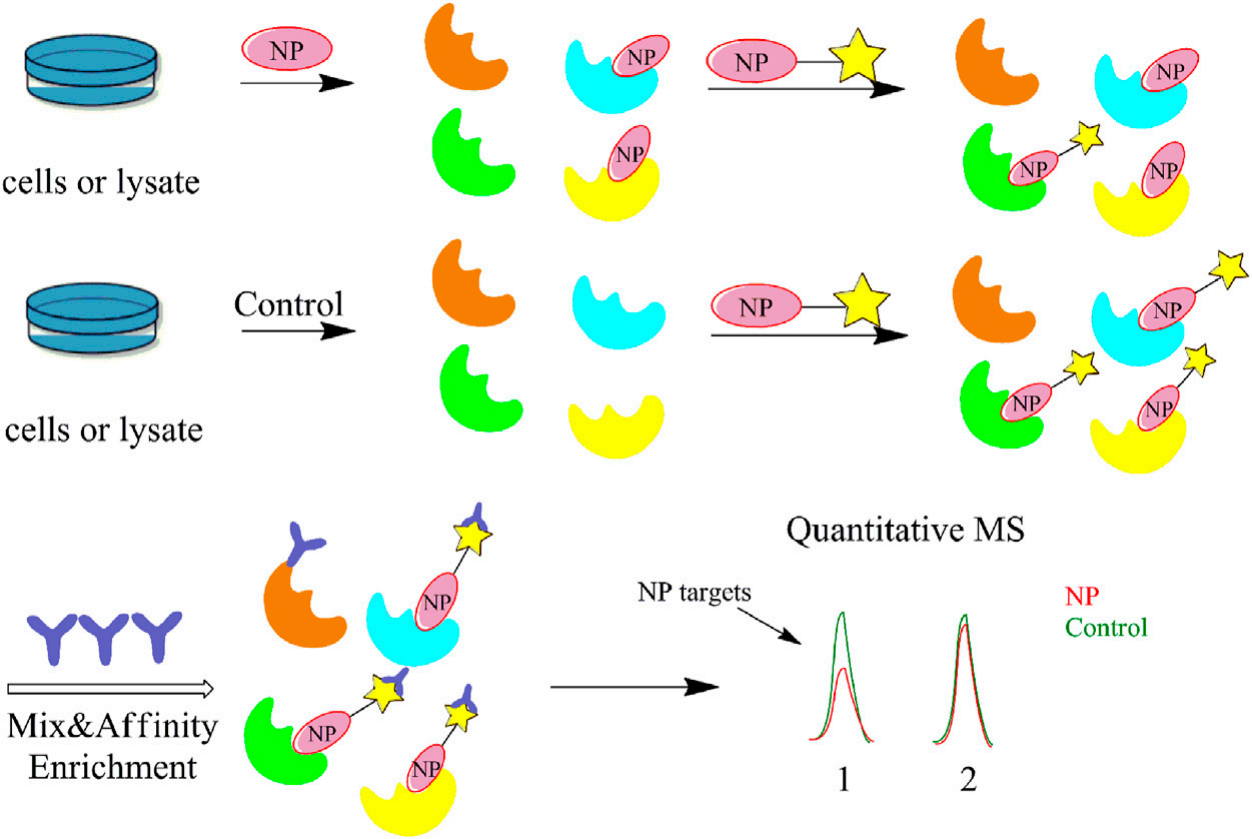
Flow diagram of the competitive activity-based protein profiling (ABPP) strategy. It allows the precursor compound of the probe to be co-incubated with the proteome before adding the probe to bind with the protein. Then, it is possible to obtain the true target protein by comparing the protein and active site labelled by the probe before and after the addition of the precursor compound.

The greatest limitation of the CCCP and ABPP methods is the non-specific binding of the probe to the protein, which often gives false positive results and makes it difficult to remove interference from highly abundant and viscous proteins. In contrast, competitive ABPP allows the precursor compound of the probe to be co-incubated with the proteome before adding the probe to bind with the protein. Hence, it is possible to obtain the true target protein by comparing the protein and active site labelled by the probe before and after the addition of the precursor compound, greatly reducing the interference of non-specific proteins in the experiment (Figure 5). Many research groups have already screened the target proteins and potent enzyme inhibitors of several NPs using this method, including inhibitors of human α/β-hydrolase domain containing 11 (ABHD11) (Navia-Paldanius et al., 2016), celastrol (Zhou et al., 2016b) and withaferin A (Grossman et al., 2017). Wang et al. used a competitive ABPP approach to test the selective binding proteins of different fluorophore probes and found that probes targeting the prostate-specific membrane antigen (PSMA) can be potentially developed as contrast agents for clinical fluorescence-guided intraoperative procedures (Wang X. et al., 2014). The competitive ABPP strategy can synthesize probes for low-abundance and structurally complex NPs, but has certain drawbacks. For example, the probe species are mostly composed of several specific active amino acid residues or protein families,.and the competitive ABPP technique is difficult to perform in a high-temperature superconducting environment. Hence, the scope of its application needs to be further explored. To overcome this limitation, Cravatt’s group developed the fluorescence polarization (fluopol)-ABPP method to create a high-throughput competitive screening platform that can also study enzymes with unknown substrates (Deng et al., 2020), while Wirth et al. identified acetaldehyde dehydrogenase as the specific target of duocarmycin in A549 cancer cells by (fluopol)-ABPP (Wirth et al., 2012).

#### Isotope Tandem Orthogonal Proteolysis-Activity-Based Protein Profiling

**FIGURE 6:**
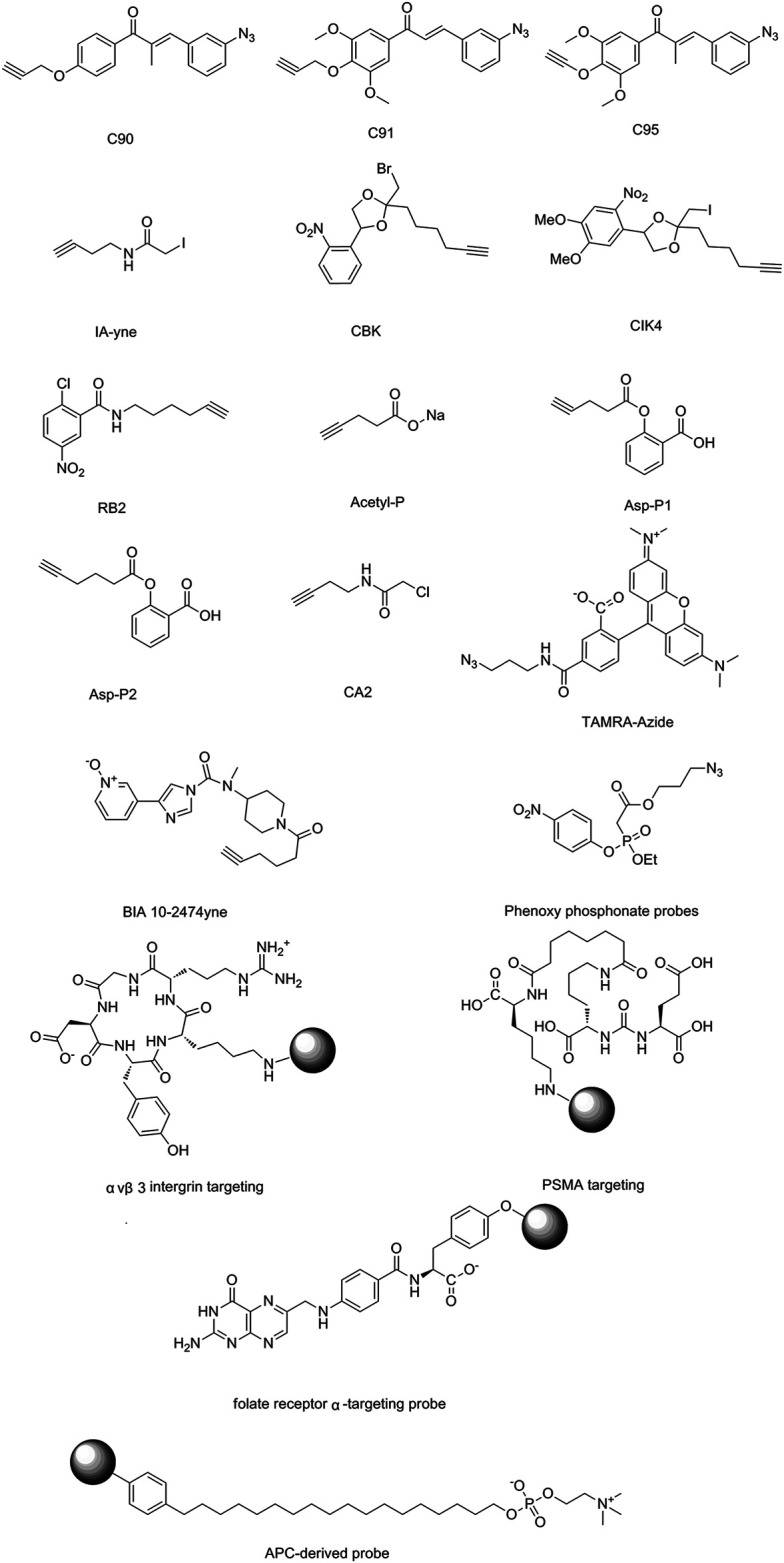
Structures of ABPP and CCCP probes for bioactive natural products.

The isoTOP-ABPP method involves the labelling of cysteine residues for enrichment using an iodoacetamide (IA) isotope-labelled probe with an alkyne stalk. Cysteine is an important nucleophilic amino acid that often influences the biological activity and pharmacological effects of NPs. Thus, active cysteine residues, which are commonly targeted by covalent inhibitors, are vital binding spots for the potential inhibition of protein activity and function in the development of new drugs. Weerapana et al. developed a set of IA isotope-labelled probes, namely the IA-light and IA-heavy probes, that was simple to synthesize and allows the quantitative analysis of proteins (Abo et al., 2018). This probe set was used to assess cysteine reactivity in purified thioredoxin and in complex proteomes, providing an alternative strategy for monitoring cysteine reactivity. Importantly, these isotope-labelled probes may also be used to quantify the percentage of cysteine modifications in individual samples. There has been innovation in the types of probes available, evolving from tags labelled with isotopes and cleaved by proteases into chemically cleavable (Qian et al., 2013; Qian and Weerapana, 2017) and photocleavable (Szychowski et al., 2010) tags. As probes and linkers continue to be developed and mass spectrometers and data analysis software are upgraded, the number of identified cysteines will increase, and their targets and functions will become clearer (Maurais and Weerapana, 2019). Notably, Weerapana and Wang have collaborated for the development of the reductive dimethyl tandem orthogonal proteolysis (rdTOP)-ABPP technique that can simultaneously identify the target proteins and their specific binding sites, while providing both quantitative detection and analytical capabilities (Yang et al., 2018). In addition, the quenched near-infrared fluorescent (qNIRF)-ABPP was developed for monitoring chemotherapy response and early diagnosis *in vivo* (Garland et al., 2016). For instance, Abd-Elrahman et al. synthesized the burst probe GB137 and non-burst probe GB123 for determining the distribution of histone proteases in *in vivo* models of atherosclerosis (Abd-Elrahman et al., 2016), while Wang et al. invented the quantitative acid-cleavable (QA)-ABPP method to identify both the target proteins and the peptides after protein hydrolysis for the molecular targets of aspirin (Wang et al., 2015). Thus, these approches will provide new directions and impetus for drug development as the technology progresses (Maurais and Weerapana, 2019). The structures of the ABPP probes used in previous studies are shown in Figure 6 (Prothiwa et al., 2016; Brøsen et al., 2017).

### Assistive Technology for Target Identification

In recent years, the rapid development of new biochemical techniques and instruments has also opened up more possibilities for target identification. The combination of quantitative mass spectrometry and chemical probe approach can significantly improve the efficiency and accuracy of NPs target identification. Currently, the most commonly used quantitative mass spectrometry techniques include stable isotope labeling by amino acids in cell culture (SILAC) and isobaric tags for relative and absolute quantification (iTRAQ).

#### Stable Isotope Labeling by Amino Acids in Cell Culture

**FIGURE 7:**
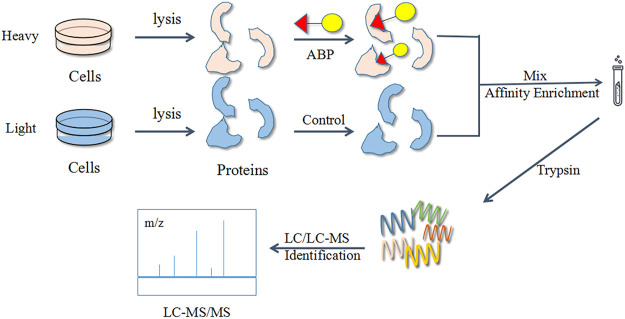
Flow diagram of the combined activity-based protein profiling (ABPP) and stable isotope labeling by amino acids in cell culture (SILAC) strategies. Firstly, it utilizes a set of amino acid isotope markers for two cell populations to be cultured. The probe is added to the heavy group and the light group is used as a control group. The labelled proteins are analyzed and identified by MS against normal proteins after a period of time.

The stable isotope labeling by amino acids in cell culture (SILAC) technique is a popular choice for quantitative ABPP studies. Numerous studies have used a combination of the ABPP and SILAC methods for target identification and exploration of bioactive functions (Figure 7). Generally, SILAC technique is used for living cells with active metabolism, avoiding errors (e.g., mutations) during experiments. However, this method is largely limited by the efficiency of metabolic activity and not suitable for primary cells and tissues (Wang S. et al., 2018).

SILAC utilizes a set of amino acid isotope markers for two cell populations to be cultured. The labelled proteins are analyzed by MS against normal proteins after a period of time. Using this method, Li et al. identified multiple protein targets of eupalmerin acetate in HL-60 cells, reflecting its properties (Li et al., 2013), while Liao’s group identified cysteine 140 as the site of sappanone for the selectively inhibited inosine 5′-monophosphate dehydrogenase type II (IMPDH2), which effectively suppressed the neuroinflammatory response (Liao et al., 2017). Additionally, Brisdelli’s team validated the change of eight proteins in quercetin-treated K562 cells (Brisdelli et al., 2020), while Yang et al. expanded the known target proteins of 4-hydroxy-2-nonenal (HNE) by an order of magnitude (Yang et al., 2015). Zhuang et al. combined ABPP with SILAC-based quantitative proteomics to identify and quantify probe-labelled protein targets by liquid chromatography–tandem MS (LC–MS/MS) (Zhuang et al., 2017). The experiments focused on the treatment of light and heavy HeLa cells separately using a photoaffinity probe, with the light cells irradiated under UV light while the heavy cells were not UV cross-linked. The light and heavy cells were collected and lysed, and their proteomes were mixed in a 1:1 ratio with CuAAC and ligated to the azide biotin marker. After streptavidin enrichment and trypsin digestion, the digested peptides were analyzed by LC–MS/MS. The SILAC ratio of each protein was quantified, excluding all targets due to non-specific binding to streptavidin, and the remainder were specific “probe-bound” proteins. The team has successfully identified over 600 bile acids (BAs)-interacting protein targets, including known bile acids (BAs) endogenous receptors and transporter proteins. In addition, the ABPP–SILAC strategy identified the target proteins for callyspongynic acid (Nickel et al., 2015) and zerumbone (Kalesh et al., 2015).

#### Isobaric Tags for Relative and Absolute Quantification

The iTRAQ method, a chemical approach innovated from SILAC, is used to add a control probe to the original probe, followed by co-culturing with live cells or cell lysates. Then, the labelled proteins are enriched and hydrolyzed with a suitable iTRAQ reagent selected for MS. Unlike SILAC, the iTRAQ technique can analyze eight samples in one LC/MS run. Using this method, pre-treated natural *Aspergillus fumigatus* G-13 fermented lignocellulose substrate was found to have a strong effect on lignin-degrading enzyme activity and protein expression (Li et al., 2021). Xia et al. identified 6,072 proteins and discovered that astragaloside IV can inhibit the invasion of cervical cancer cells with the induction of their autophagy (Xia et al., 2020). Additionally, the combination of ABPP and iTRAQ revealed a series of target proteins, such as andrographolide (Wang J. et al., 2014) and curcumin (Wang et al., 2016), as well as their binding mechanisms. Furthermore, Nishino et al. treated four samples of lysis products containing different hypromellose concentrations for 30 min and then added suitable probes for co-culture (Nishino et al., 2013). Under these conditions, the covalently modified proteins were coupled to biotin azide for further affinity purification and elution. Each sample was later derivatized with the unique iTRAQ reagent, and the mix was used for fractionation and MS analysis. This experiment identified peptides corresponding to 10 protein kinase and revealed TbCLK1 as a therapeutic target for African trypanosomiasis.

## Non-Probe Approach

### Biophysical Methods

The chemical probe approach of identifying NPs targets has been described in detail in the previous section. The limitations of this approach are determined by the single modification site of the NPs, lack of synthetic methods, and necessary modification of the NPs during the experiment, which may cause the alteration or loss functional activity and thereby failing the identification of the true target protein. Hence, these factors have greatly hindered the research on NPs and their applications (Li et al., 2019b). However, the advent of biophysical methods for target identification has compensated for these deficiencies. Since most proteins fold into their natural conformation through intramolecular non-covalent interactions, then the interaction of NPs with the target proteins can alter their structure and stability. Therefore, the true target protein can be identified by detecting the difference in protein changes before and after the addition of the ligand compound. Biophysical methods can also detect direct-acting proteins and possibly indirect-acting proteins without modifying the NPs, providing another direction for unravelling the mechanisms of action of important NPs.

#### Drug Affinity Responsive Target Stability

**FIGURE 8:**
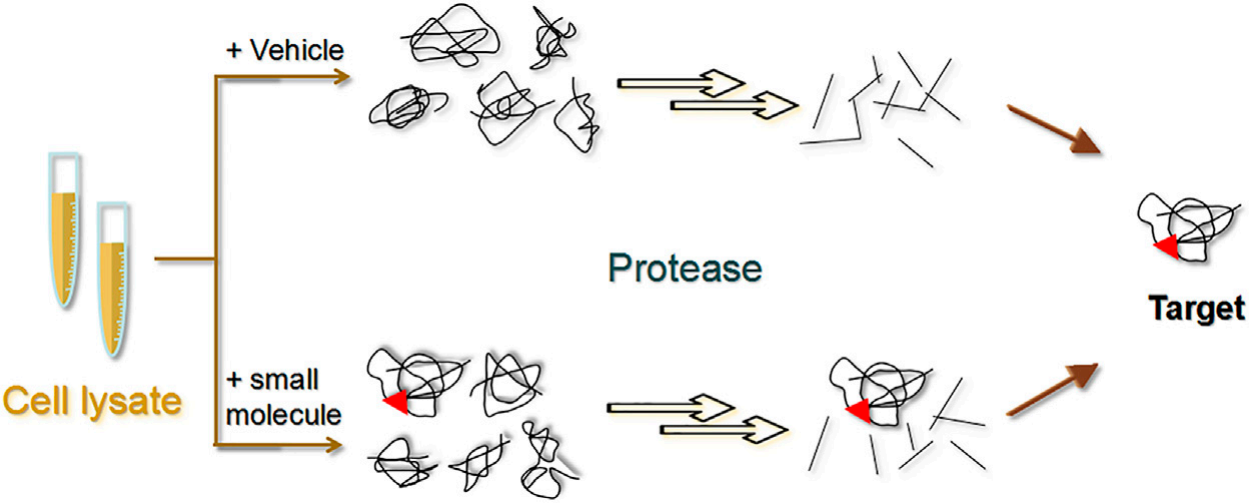
Schematic diagram of the drug affinity responsive target stability (DARTS) strategy. The experiment is mainly divided into a small molecule group and a control group. Target proteins bound to small molecules are not readily hydrolysed by proteases.

DARTS was first proposed in 2009 by Lomenick et al. as a method to recognize the small molecules of target proteins without modifying the corresponding NPs (Lomenick et al., 2009). Specifically, the ligand binds to the target protein to form a stable protein conformation that is not easily hydrolyzed by proteases (Figure 8) (Lomenick et al., 2009). Several targets for NPs, such as resveratrol (Lomenick et al., 2009) and rapamycin (Lomenick et al., 2009), have been discovered using this approach.

The DARTS technique can be used to find the target of a compound based on the histological changes in the proteins between treated and untreated samples (Zhao et al., 2020). Proteins are separated by SDS-PAGE to visualize the conserved bands and then analyzed by LC–MS. Gum staining techniques and two-dimensional electrophoresis may also be used for detection. In 2012, Tohda et al. found that object recognition memory was significantly improved in diosgenin-treated 5XFAD mouse. Furthermore, 1,25D_3_-membrane-associated rapid response steroid-binding protein (1,25D_3_-MARRS) was identified to be a target of diosgenin using DARTS (Tohda et al., 2012). In 2017, Yang’s team discovered that bone marrow tonic exhibited enhanced memory function and improved AD pathological changes in 5XFAD mice (Yang et al., 2017). Additionally, disintegrin response mediator protein 2 was identified as a target of naringin using a combination of DARTS and LC–MS (Yang et al., 2017). In 2018, Ge et al. isolated an ergosterane-type steroid compound from spiders (Araneae) named aminosteroid D that was found to act on pyruvate kinase muscle isoform 2 (PKM2)—the rate-limiting enzyme of glycolysis in host cells—which suppresses HIV replication, and thus, inhibits HIV proliferation (Ge et al., 2018). Furthermore, Cassiano et al. identified several targets for the natural bioactive compound magnolol (Cassiano et al., 2019). In brief, unmodified magnolol was selected and co-incubated with samples of HeLa cell lysates, followed by limited protein hydrolysis with *Bacillus subtilis* protease. The DARTS and SDS-PAGE experiments revealed a direct interaction between magnolol and importin β1 (Cassiano et al., 2019).

One of the greatest advantages of the DARTS method is that chemical derivatization is not needed when using natural small molecules, and knowledge of the chemical nature and purity of the compounds is not required. As a result, DARTS allows the biologically active NPs to be used for targeted isolation, allowing studies beyond herbal pharmacology. Nevertheless, the limitations are obvious, such as the usually high level of non-specific binding of non-target proteins to the matrix, making the isolation of the true target protein more difficult. Although extensive washing can help reduce the amount of impurities, the target proteins will also be lost during the washing process (Lomenick et al., 2011).

#### Stability of Proteins From Rates of Oxidation

**FIGURE 9:**
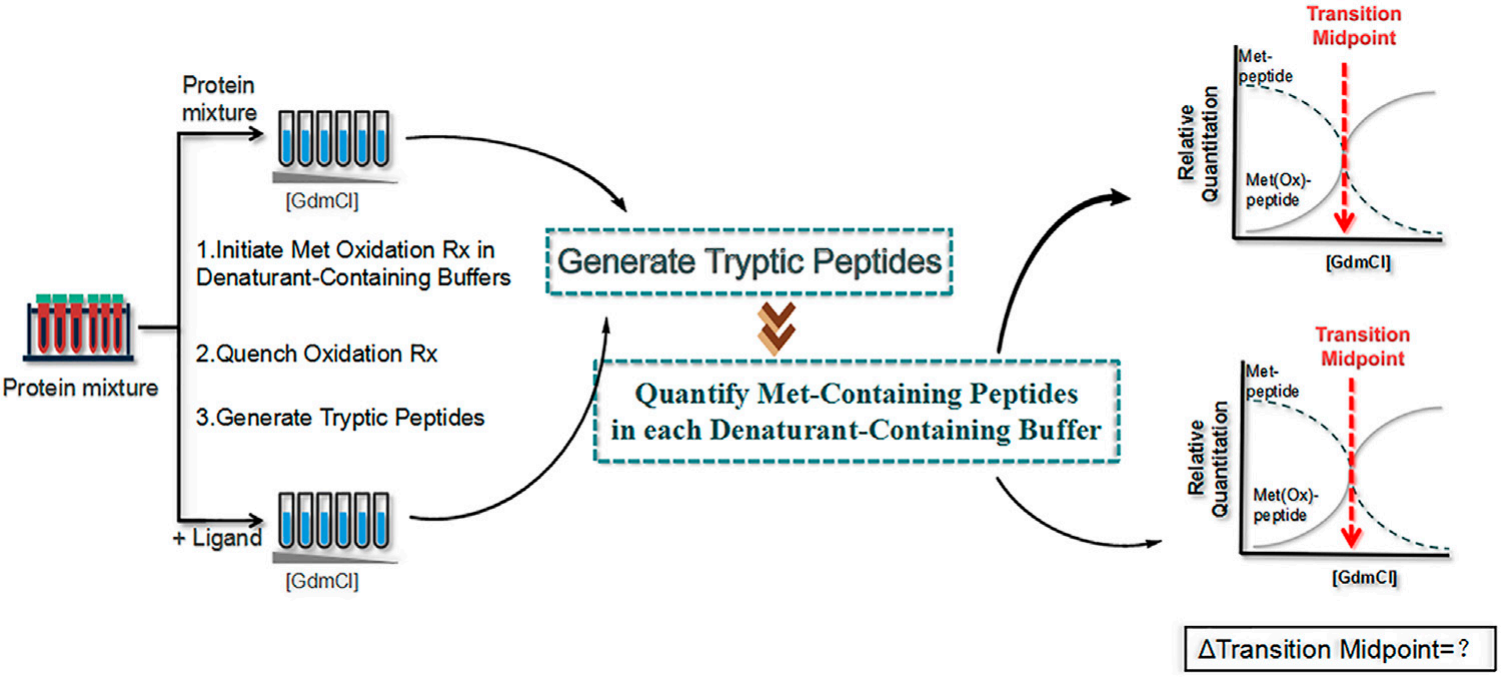
Schematic diagram of the stability of proteins from rates of oxidation (SPROX) strategy. First, two protein samples (with and without ligand) are dispensed into a buffer containing a chemical denaturant , and hydrogen peroxide is added to the protein sample. Then, the oxidation reaction is quenched with an excess of methionine, and the protein sample is precipitated with tricarboxylic acid for subsequent quantitative proteomics to obtain the oxidation ratio of oxidized methionine.

SPROX (Hughes et al., 2009) is a new method proposed in 2010 that measures the level of methionine oxidation of the target protein instead of detecting the pattern of protein hydrolysis (Figure 9). First, the protein sample (with or without ligand) is dispensed into a buffer containing a chemical denaturant to bring the protein into folding–unfolding equilibrium, which is analogous to the first step in a pulsed protein hydrolysis method. Then, hydrogen peroxide is added to the protein sample to react with the methionine side chain of the protein. Finally, the oxidation reaction is quenched with an excess of methionine, and the protein sample is precipitated with tricarboxylic acid for subsequent quantitative proteomics to obtain the oxidation ratio of oxidized methionine. The addition of the drug leads to the increased structural stability of the target protein, which in turn reduces methionine exposure and oxidation.

Using SPROX, the targets of action of the immunosuppressant cyclosporin A were identified in yeast lysates. These included two known target proteins, procyclosporin A and UDP-glucose-4-epimerase, and eight new target proteins, including carbamoylphosphate synthetase, glycogen synthase, and glutamate dehydrogenase (West et al., 2010). In addition, six new targets of the resveratrol were identified by Dearmond’s group using SPROX (Dearmond et al., 2011). Wallace et al. applied iTRAQ–SPROX for the large-scale analysis of protein–ligand binding interactions and successfully analyzed >1,100 proteins. Notably, filamin A and elongation factor 1α were identified as important targets of manassantin A in hypoxic cells (Geer Wallace et al., 2016).

However, the main disadvantage of SPROX is that it is limited to the identification and accurate quantification of the most abundant proteins only in each sample. Furthermore, only methionine-free peptides are useful for SPROX analysis and not all methionine residues exhibit different rates of oxidation, which cannot provide sufficient information for the conclusive identification of the NPs ligands interacting with target proteins.

#### Cellular Thermal Shift Assay

In addition to focusing on the enzymatic and oxidative stability of the target protein, its thermal stability can also be examined. The degradation temperature and trend of the target protein can be used as an important indicator to distinguish the target protein from other proteins. Previous studies combined the thermal stability of gel electrophoresis and immunoblotting to analyze the drug-specific target binding for CETSA (Chen, 2020). In 2018, Wang and colleagues combined CETSA, molecular docking, and cell-based assay validation and identified nucleolin (NCL) as a target of curcumol that can inhibit the progression of nasopharyngeal carcinoma (Wang J. et al., 2018). In the same year, Vasaturo’s group used a combination of proteomics, CETSA and classical biochemical techniques to demonstrate that the interaction of oridonin with NCL can effectively modulate the activity of heat shock protein 70 (HSP70) (Vasaturo et al., 2018). In 2019, Anette et al. established a multi-group microtubule protein-specific CETSA technique to reveal the anticancer activity of paclitaxel, which binds to the β-microtubulin on the luminal side (Langeback et al., 2019). Guo’s group extracted a derivative from *Aspergillus flavus* that was selectively toxic to phosphoglycerate dehydrogenase (PHGDH)-dependent cancer cells. The derivative was confirmed to bind directly to PHGDH using microscale thermophoresis (MST) and CETSA (Guo et al., 2019). In addition, Tu’s and Zeng’s teams combined CETSA and SILAC to identify the targets of protocatechualdehyde (PCA) affecting myocardial fibrosis as type I collagen (Wan et al., 2019).

The advantage of CETSA is that intact cells are used and no treatment or preparation is required. It is also very selective due to the Western blot analysis step. However, some target proteins with unexpanded binding sites may not be detected. Additionally, some of the antibodies used for Western blotting are non-specific, and off-target proteins may be identified as false positives (Chen, 2020). Therefore, CETSA is not suitable for highly heterogeneous proteins and proteins where unfolding of the ligand-binding structural domain does not cause aggregation and denaturation (e.g., DNA-binding and chaperone proteins) (Dziekan et al., 2019).

#### Thermal Proteome Profiling

**FIGURE 10:**
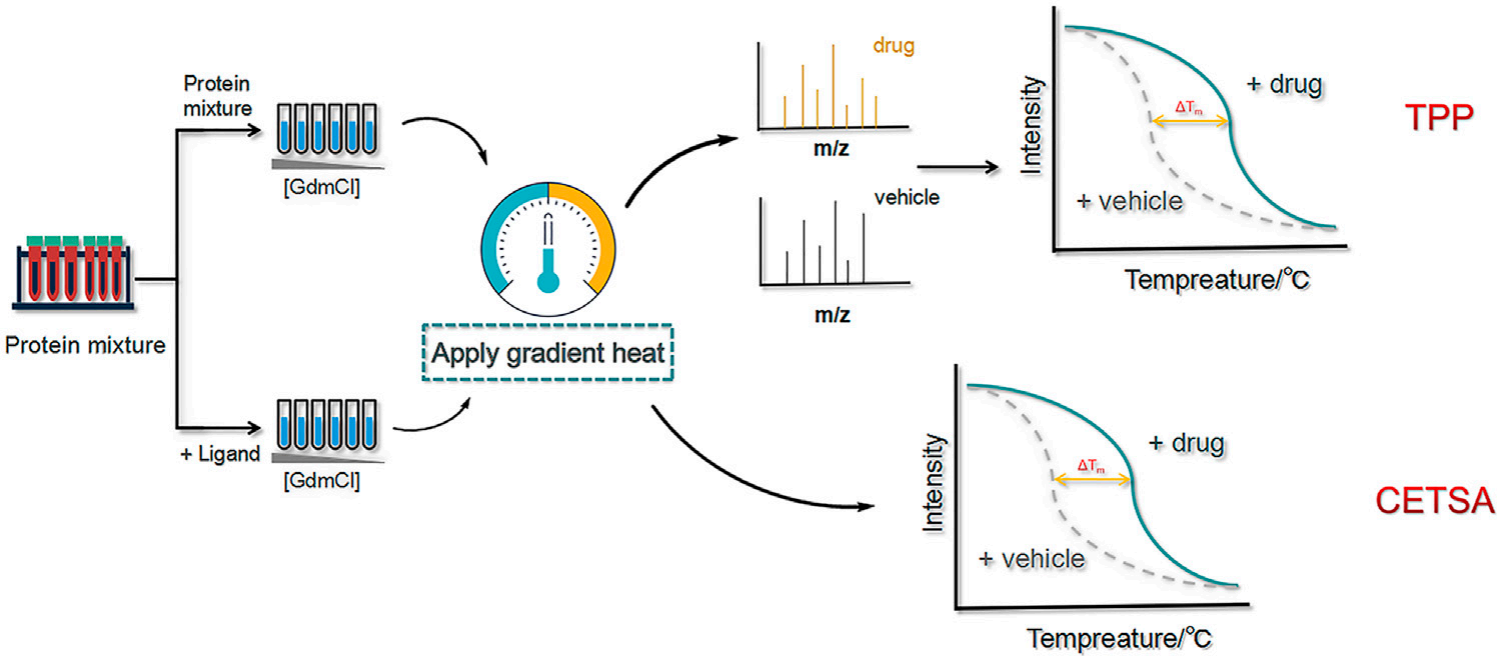
Schematic diagram of the thermal proteome profiling (TPP) and cellular thermal shift assay (CETSA) methods. First, two protein samples (with and without ligand) are dispensed into a buffer containing a chemical denaturant. Then,high-resolution MS is performed using neutron-encoded isobaric mass TMT10 as a labelling reagent, and the complete melting curves of heavily expressed soluble proteins are obtained.

As a more advanced approach than CETSA, TPP is capable of identifying proteins that exhibit ligand-induced thermal stability at higher temperatures and combining multiplexed quantitative MS to assess ligand–target engagement at the cellular level. To promote thermal stability, high-resolution MS is performed using neutron-encoded isobaric mass TMT10 as a labelling reagent, and the complete melting curves of heavily expressed soluble proteins are obtained (Figure 10).

Adhikari et al. assayed protein–ligand binding interactions using proteins from yeast cell lysates. The tight binding interactions between cyclosporin A and cyclophilin A were successfully detected and quantified in replicate analyses (Adhikari and Fitzgerald, 2014). Kirsch et al. discovered nucleoprotein 14 was the target protein of vioprolide A derivative from Jurkat cells by TPP (Kirsch et al., 2020). In 2020, Lyu et al. proposed a microparticle-assisted precipitation screening (MAPS) method for the analysis of insoluble precipitates (Lyu et al., 2020). With the aid of the particles, sample loss was minimized and sample preparation was simplified. MAPS was previously used to successfully identify several drug targets, including 32 protein kinases of astrosporin that were screened from 20 μg of initial protein sample (80% specificity).

TPP is a broad-spectrum protein identification technique that has good stability, can identify numerous proteins, and does not require incubation with antibodies. However, it is time-consuming, costly, has limited detection of membrane proteins, and has a high probability of false positive results; thus, the method needs further improvement.

### Computational Prediction

Computer simulations using chemical biology data provide an alternative to laboratory experiments for target identification (Chen, 2016). Screening compounds in protein databases allows the identification of candidate targets for a particular compound and facilitates the manipulation of subsequent experiments. In particular, it is used to make more flexible and computationally inexpensive predictions of ligands with remarkable predictive performance, which has come a long way in the last decade and continues to evolve (Yang S. Q. et al., 2020).

In the future, the use of quantum computing, computational software, and public databases to model molecular interactions and to predict the characteristics and parameters required for developing new drugs, such as pharmacokinetics and pharmacodynamics, will greatly reduce false positive leads during drug development (Thomford et al., 2018). One of the challenges that scientists need to address in using big data is how to integrate the vast amount of information into a meaningful and manageable unit. To understand histological data and revolutionize clinical medicine, clinical phenotype data must be combined with the corresponding genomic, transcriptomic, proteomic, and epigenomic data.

### Omics-Based Approaches

#### Transcriptomics

Transcriptomics methods (e.g., DNA microarrays, RNA sequencing, gene editing) are technologies that rely on the establishment of sequence diversity and provide tremendous technical support for detecting the expression of RNAs of the whole genome. DNA microarrays are used to immobilize huge quantities of oligonucleotides, peptide nucleic acids, or DNA onto a very small substrate, such as silicon, slides, or nylon membranes, allowing simultaneous analysis of the effects of multiple components of an active biomolecule on multiple gene subgroups (Gu, 2004). It has the advantage of using very little material, high sensitivity, the ability to screen a larger number of genes in parallel, and the ease of comparative analysis of large numbers of samples. In addition, DNA microarrays can be used to study drug–drug interactions, characterize on- and off-target effects in the optimization of new therapeutic agents, and provide a good insight into the molecular mechanisms and networks underlying the complex pharmacological functions of bioactive NPs (Luo and Tang, 2002). However, the tests used in routine clinical practice require high-quality data, and DNA microarrays are expensive, has low design flexibility, and high diagnostic sensitivity due to the surface bound probes, which may lead to false negative results (Chiodi et al., 2021). The results obtained from microarrays should be validated by combination with other methods, such as *in-situ* hybridization, reverse transcription–polymerase chain reaction (RT-PCR).

#### Proteomics

Proteomic analysis is an approach to identify drug targets by examining the differences between proteins in cells before and after drug action. This method can detect factors that only affect protein expression and is more comprehensive than Transcriptomics. There are various methods to examine proteins, including bi-directional gel electrophoresis (Yue et al., 2008), two-dimensional LC, MS/MS, and two-dimensional difference gel electrophoresis (2D-DIGE).

Bengamides are a class of marine NPs that can inhibit tumor growth both *in vitro* and *in vivo*. Towbin et al. used two-dimensional gel electrophoresis to demonstrate that bengamides (Towbin et al., 2003) can directly or indirectly inhibit methionine aminopeptidase (MAP) by binding to the enzyme via a mimetic peptide substrate. On the other hand, Kong’s team employed a combination of iTRAQ, two-dimensional LC, and MS/MS to investigate the effect of artesunate (ART) on *S. japonicum* proteome in susceptible mice. This experiment identified multiple targets and provided the first protein expression profile of *S. japonicum* in response to ART treatment, which offered a better understanding of the molecular mechanism underlying the therapeutic action of ART (Kong et al., 2015). Garcinia cambogic acid (GA) is an anticancer drug undergoing phase IIb clinical trials in China. Yue et al. identified two new targets of GA, heat shock protein 27 (HSP27) and vientin, using comparative proteomics (Yue et al., 2016). Currently it is quite difficult to use proteomic to identify the direct target proteins of NPs. Only very few examples of direct targets can be inferred.

#### Cytology

Cellular metabolomics provides relevant information on specific cell types under different conditions to explore the nature and function of cells. This can also be used to discover the targets of action of NPs, including cell morphology analysis, cellular activity screening, and intra-cellular analysis. Not only can this technique compare the biochemical differences between healthy and diseased organisms and provide information on the primary causes of diseases, but can also reveal the end products of cellular regulatory pathways and identify potential targets for pharmacological intervention (Zhang et al., 2019).

Furthermore, Moussa et al. found that resveratrol treatment significantly enhanced the action of 116 kDa poly (ADP-ribose) polymerase, causing the nuclear fragmentation of SJSA1 osteosarcoma cells and consequently inhibiting osteosarcoma cell activity (Alkhalaf and Jaffal, 2006). Titov’s group examined the effects of tretinoin on the protein, RNA, and DNA synthesis in HeLa cells using an isotope labelling assay and determined that the molecular target of tretinoin is the XPB subunit of the general transcription factor TFIIH (Titov et al., 2011). A study on *Fusarium oxysporum*, which can produce a lignan-like tetraacid named TA-289 that can induce cell death by directly inhibiting one or more mitochondrial localization targets, revealed the molecular basis of lignan-like compound activity (Quek et al., 2013).

#### Metabolomics

Metabolites are the products of a network of intracellular enzymatic reactions that play a crucial part in various signal transduction pathways. Since the presence of metabolites can be correlated with the inactivation of specific enzymes, a targeted approach to the labelling, enrichment, and identification of individual metabolite classes is required to identify the true target of NPs and fully elucidate the properties and functions of these important metabolites. The main method of metabolomics is the discovery metabolite profiling (DMP), a type of molecular profiling of small metabolites. For instance, Sagathelian’s group identified a potential target for N-acyl taurine (NAT) as fatty acid amide hydrolase (FAAH) through DMP (Saghatelian et al., 2004). However, there are still major challenges in the field of metabolomics that are needed to be addressed, such as the lack of a rapid and reliable method that can determine the structure of identified metabolites based on large amounts of data.

### Bioinformatics

Lamb et al. constructed the CMAP Phase I reference gene based on the principle of graphical matching using a large database of signature gene expression profiles via a systematic approach to discover associations between functional diseases, genetic perturbations, and drug effects, as well as via data mining using pattern matching software (Lamb et al., 2006). The CMAP gene expression profiles were subsequently linked to compounds, genes, and disease responses, revealing compounds with similar modes of action and physiological processes and demonstrating connections between diseases and drugs. In 2017, Lv et al. stablished the first NPs small molecule gene expression profiling database platform in China that can be used in combination with CMAP to predict the pharmacological activity of small molecules, molecular targets, and associated pathways for new drug development (Lv et al., 2017). These features demonstrate both the feasibility of this approach and the great value of the large-scale linkage mapping CMAP project.

## Prospects and Conclusions

Bioactive NPs have several origins, exist widely in nature, and has excellent potential for various applications. However, only a small proportion of the currently known NPs can successfully exert their medicinal effects. Many biologically active molecules achieve their functions by interacting with protein targets. However, the targets of several NPs are still unidentified, which is a major bottleneck that hinders further research into their applications. Therefore, target identification of bioactive NPs is essential for the research of modern drugs. Not only can this elucidate the mechanisms and targets of action for developing new drugs, but also meets the constant demand for new drugs and drug precursors.

Currently, the commonly used techniques for identifying targets can be classified into two types. Chemical probe approaches are the more popularly used methods, such as CCCP and ABPP,. On the other hand, non-probe approaches identify target proteins from new perspectives to complement chemical probe approaches. In this review, the advantages and disadvantages of the currently available target identification methods are summarized in Table 2.

**TABLE 2:** The advantages and disadvantages of currently available target identification methods.

Target identification Methods	Advantages	Disadvantages
CCCP	1) Incorporates cross-cutting methods from synthetic chemistry, cell biology, and mass spectrometry.	1) Molecules with specific affinity are difficult to obtain.
	2) Employs simple synthesis and indiscriminate analysis of all adsorbed proteins.	2) Molecules with large and diverse structures are difficult to immobilize in solid-phase carriers while retaining their activity.
ABPP	1) Probe synthesis is easy and does not require very laborious steps.	1) Only specific proteins present in the cell can interact with the compound.
	2) Reduces the impact of probe synthesis on the structure and activity of the original natural product.	2) Most experiments are performed *in vitro* using cell lysates, which do not fully simulate the physiological conditions in cells *in vivo*.
DARTS	The use of natural small molecules does not require chemical derivatization or knowledge of the chemical nature and purity of the compound, allowing studies beyond pharmacology and herbal pharmacology.	1) Some compounds do not produce significant conformational changes when bound to their targets.
		2) Some proteins have low overall sensitivity to protein hydrolases and do not produce detectable changes.
		3) Some target proteins have increased hydrolytic sensitivity upon binding to the compound.
		4) Non-specific binding of non-target proteins to substrates is often high.
SPROX	1) Enables large-scale assessment of protein folding states.	1) Not suitable for the detection of insoluble proteins.
	2) Allows precise measurement of the structural domains and peptides bound to the target protein by the compound.	2) Only proteins and ligands with high concentrations can be detected.
	—	3) May interfere with protein folding properties and ligand binding.
	—	4) The procedure is complex, expensive, and requires a lot of consumables.
CETSA	Uses intact cells, requires no treatment or preparation, and is very selective.	1) Some target proteins with unfolded binding sites may not be detected.
		2) Not applicable to highly heterogeneous proteins and proteins where unfolding of the ligand-binding domain does not cause aggregation and denaturation.
TPP	1) Has good stability and a high number of proteins can be identified.	1) Time consuming and costly.
	2) Incubation with antibodies is not required.	2) Limited detection of membrane proteins and target proteins with low abundance.
	3) A broad-spectrum protein identification technique.	3) Low thermal stability.
	—	4) High probability of false positive results.
Computational prediction	1) Potential candidate targets suitable for that particular compound can be identified, facilitating subsequent experiments.	Integrating large amounts of information into a meaningful and manageable unit is difficult.
	2) Prediction of ligands is more flexible, computationally inexpensive, and has high-throughput performance.	
Transcriptomics	Identification is more reliable and sensitive, enabling more genes to be screened in parallel, and facilitating comparative analysis of large numbers of samples.	1) Very expensive and has low design flexibility.
		2) Surface binding probes can affect diagnostic sensitivity and lead to false negative results.
Proteomics	Factors that affect only protein but not gene expression can be detected, making analysis more comprehensive than Transcriptomic methods.	1) Procedures are costly to perform.
		2) Effectiveness is susceptible to variation depending on the type of protein.
Cytology	Capable of qualitative and quantitative analysis of endogenous small molecules to reveal the relationship between different pathways in living cells.	1) Cannot give direct information on target proteins.
		2) Not widely applicable.
Bioinformatics	Integrating gene expression profiles to compounds, genes, and disease responses can also be used for drug development.	The amount of work required to set up a CAMP platform is greater and more difficult.

CCCP, compound-centered chemical proteomics; ABPP, activity-based protein profiling; METPR, metabolite enrichment by tagging and proteolytic release; DARTS, drug affinity responsive target stability; SPROX, stability of proteins from rates of oxidation; CETSA, cellular thermal shift assay; TPP, thermal proteome profiling.

In chemical probe approach, CCCP modifies the structure of active NPs by incorporating various reporter moieties. As a result, the ability of the NPs to identify its target in the complex cellular proteome can be improved, and information about the target protein can be more easily obtained. However, CCCP is usually performed *in vitro* and is susceptible to altered activity, which often makes it difficult to accurately reflect the intrinsic link between protein and organismal function. In contrast, ABPP is a well-established and stable method for identifying target proteins mainly used in conjunction with various advanced techniques, including CC-ABPP, PAL-ABPP, competitive ABPP, and isoTOP-ABPP. Probe synthesis for ABPP is easy and does not require very tedious steps, reducing the impact of probe synthesis on the structure and activity of the NPs. The uses of ABPP has now expanded from drug target identification to new drug discovery, laying the groundwork for future research into the interactions of NPs and higher organisms.

Notably, CCCP and ABPP focus more on the structure of the NPs itself, while other methods modify the NPs according to the binding mode of the NPs and its target protein. For complexes obtained by covalent binding, reporter groups such as biotin and fluorescently labelled probes are introduced, while less stable complexes bound non-covalently are converted into covalent linkage using photoaffinity markers to facilitate enrichment and purification. However, the biological activity of the NPs must be preserved during these modifications. Therefore, the introduction of small and non-toxic moieties that do not destroy key potent groups without the effects of spatial blocking is required, which greatly limits the practical application of such methods.

In recent years, the development of non-probe approach has been continuous. Compared to chemical probe approach, biophysics has a stricter means of identifying target proteins by utilizing the stability of ligand–drug binding to find the targets without affecting the functional activity of the NPs. However, biophysical methods can only be applied to limited targets and cover a narrow range of protein types, content, and homogeneity with highly specific requirements. In addition, biophysical methods have a higher probability of false positive results and incur higher costs. On the other hand, computational prediction using chemical biology data has a remarkable predictive performance and is continuously evolving in this era of big data. Computer simulations can compensate for the lack of diverse laboratory data but may possess inaccuracies; there have been instances where target compounds are highly similar in reference libraries. Therefore, the generation of abundant, high-quality, and more diverse proteomic databases is required to address this problem. In addition, a more accurate quantitative analysis of the cell as a whole was made possible by the rapid advances in histological and imaging techniques. As a result, new techniques like differential screening using transcriptomics probes, differential proteomics screening, and cell morphology comparison have emerged as powerful tools for the systematic study of biochemical processes during drug trials.

During target identification experiments, validation is necessary using a combination of *in vitro* binding assays, such as western blotting, immunofluorescence staining, and Försters resonance energy transfer (FRET) microscopy. The associated pathways of the identified target proteins are also critical and must be studied to obtain highly meaningful information. Therefore, this comprehensive overview of available experimental methods for target identification was necessary. With the continuous progress of science and technology, the intersection of multidisciplinary theories and the joint use of several technologies will become the main trend in the future. Notably, with the development of artificial intelligence, this trend will become more obvious and even play a decisive role in future scientific research. Thus, the integration of chemical proteomics with biophysics, transcriptomics, bioinformatics, and other disciplines will allow the improvement of currently available methods for target identification and new drug development. Finally, new and improved methods that can adapt to the complex properties of active NPs must be developed for the advancement of chemical and biological research for medicinal applications.
